# 8. Sepsis-Associated Acute Kidney Injury and Acute Kidney Disease: A 15-Year Cohort Study of 4,226 Adult Sepsis Inpatient Survivors at a Tertiary Medical Center in Taiwan

**DOI:** 10.1093/ofid/ofab466.008

**Published:** 2021-12-04

**Authors:** Chih-Chia Liang, Hung-Chieh Yeh, Pei-Shan Chen, Chin-Chi Kuo, Hsiu-Yin Chiang

**Affiliations:** 1 China Medical University Hospital, Taichung City, Taichung, Taiwan; 2 Big Data Center, China Medical University Hospital, Taichung City, Taichung, Taiwan

## Abstract

**Background:**

Sepsis is the most common cause of acute kidney injury (AKI) and about one-third of patients with sepsis-associated AKI (SA-AKI) develop acute kidney diseases (SA-AKD) and may progress to unfavorable outcomes. We aimed to study the characteristics and outcomes associated with SA-AKI and SA-AKD.

**Methods:**

This cohort study included adult inpatients with first-time sepsis who were admitted during 2003-2017, had qualifying serum creatinine (SCr) measurements at baseline (-365 to -3 days), -2 to +7 days, and +8 to +90 days of sepsis index day, and survived the first 90 days (**Figure 1**). Sepsis was identified using an electronic medical records-based Sepsis-3 criteria. We classified sepsis inpatients into SA-AKI(-), SA-AKD(-), SA-relapsed-AKD, and SA-nonrecovery-AKD (**Figure 2**). ESRD and mortality were ascertained by linking to the Catastrophic Illness records and to National Death Registry, respectively. Multivariable Cox proportional hazard model was used to evaluate the risk of mortality and end-stage renal disease (ESRD) associated with SA-AKI/AKD subtypes.

Figure 1. Flowchart of the selection process of adult sepsis survivors (N = 4226 patients).

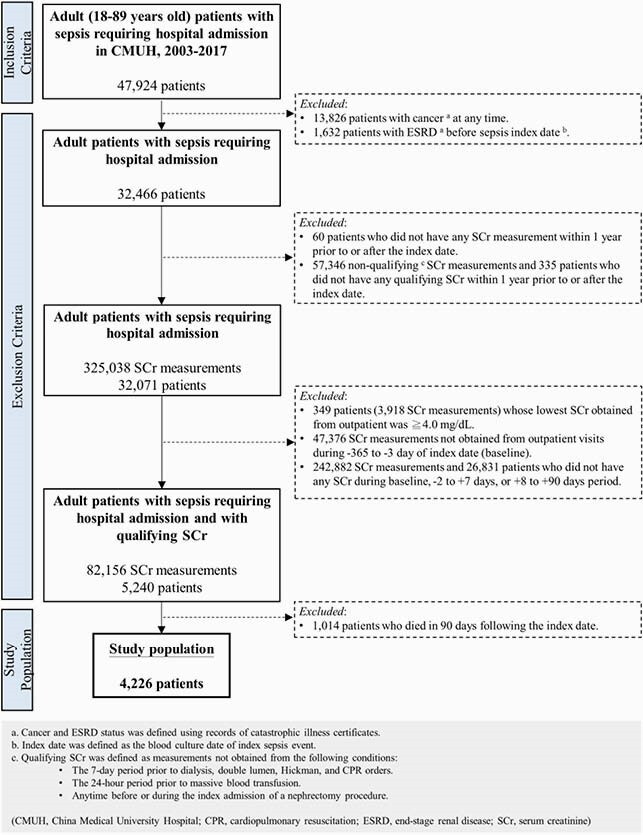

Figure 2. Definitions of sepsis associated-acute kidney injury (SA-AKI) and sepsis associated-acute kidney disease (SA-AKD).

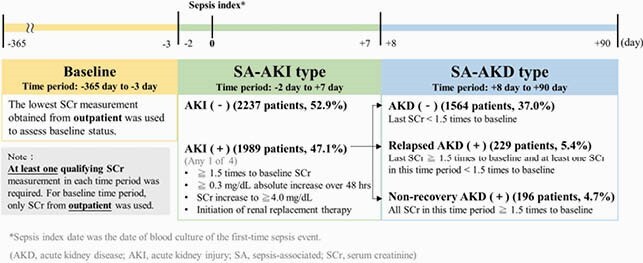

**Results:**

Of 4,226 eligible sepsis inpatient survivors, 47.1% developed SA-AKI and 10.1% progressed to SA-AKD (5.4% relapsed and 4.7% nonrecovery). Patient with AKI and non-recovered AKD had the worst baseline renal function (SCr, 1.3 mg/dL) (**Table 1**). The multivariable analyses revealed that SA-relapsed AKD was significantly associated with increased risk of all-cause mortality for 1-year (aHR 1.67; 95% CI 1.25, 2.24), 3-year (aHR 1.38; 95% CI 1.11, 1.71), and overall (aHR 1.35; 95% CI 1.12, 1.61), compared with SA-AKI(-). SA-relapsed AKD and SA-nonrecovery AKD were both significantly associated with 1-year, 3-year, and overall ESRD, with the risk of about 4-fold or higher than SA-AKI(-) (**Table 2**).

Table 1. Baseline characteristics and outcomes among adult sepsis survivors, by different SA-AKI/AKD subtypes.

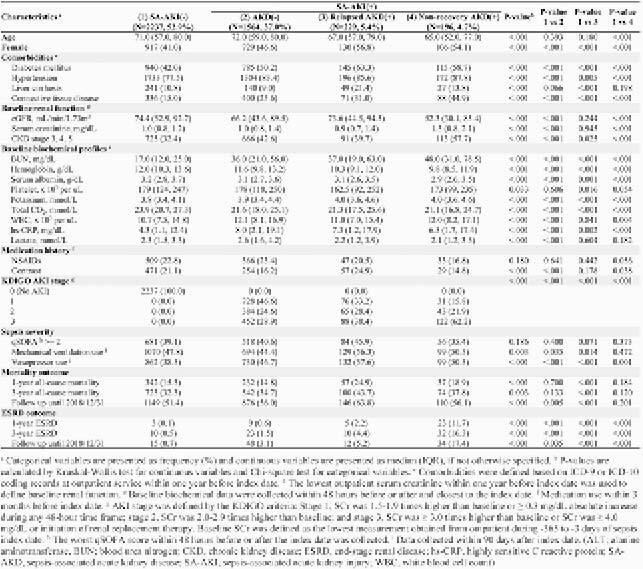

Table 2. Risk of all-cause mortality and end stage renal disease (ESRD) among adult sepsis survivors.

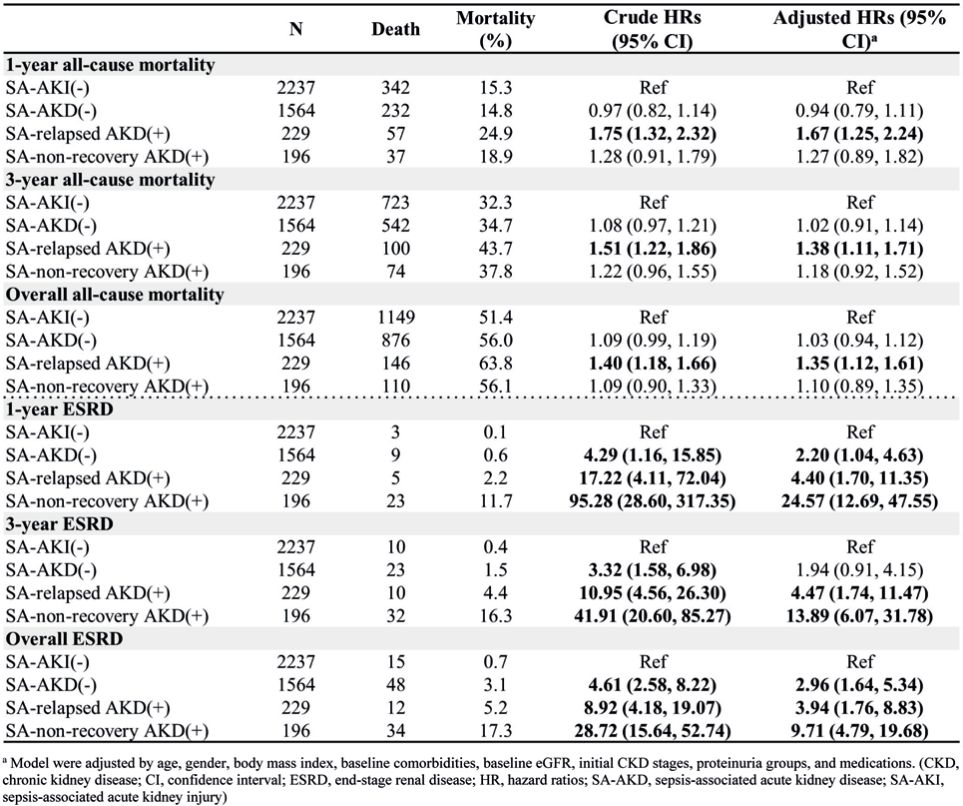

**Conclusion:**

Sepsis survivors who initially had AKI and developed relapsed or nonrecovery AKD tended to have worse outcomes of all-cause and ESRD, compared with those without AKI. Unexpectedly, patients with non-recovered AKD did not have a higher mortality risk, possibly because we have selected those who survived the first 90 days of sepsis. We will develop two-stage prediction models to identify sepsis patients at risk of developing AKI and SA-AKI patients at risk of developing different types of AKD.

**Disclosures:**

**All Authors**: No reported disclosures

